# A Mouse Intra-Intestinal Infusion Model and its Application to the Study of Nanoparticle Distribution

**DOI:** 10.3389/fphys.2016.00579

**Published:** 2016-11-29

**Authors:** Ana Sadio, Ana L. Amaral, Rute Nunes, Sara Ricardo, Bruno Sarmento, Raquel Almeida, Hidekazu Tsukamoto, José das Neves

**Affiliations:** ^1^Instituto de Investigação e Inovação em Saúde, Universidade do PortoPorto, Portugal; ^2^Institute of Molecular Pathology and Immunology of the University of PortoPorto, Portugal; ^3^Faculty of Medicine of the University of PortoPorto, Portugal; ^4^Gastroenterology Department, Unidade Local Saúde da GuardaGuarda, Portugal; ^5^Gulbenkian Programme for Advanced Medical EducationLisbon, Portugal; ^6^Instituto de Engenharia Biomédica, Universidade do PortoPorto, Portugal; ^7^Instituto de Ciências Biomédicas Abel Salazar, Universidade do PortoPorto, Portugal; ^8^CESPU - Instituto de Investigação e Formação Avançada em Ciências e Tecnologias da Saúde, Instituto Universitário de Ciências da SaúdeGandra, Portugal; ^9^Biology Department, Faculty of Sciences of the University of PortoPorto, Portugal; ^10^Department of Pathology, Southern California Research Center for ALPD and Cirrhosis, Keck School of Medicine of the University of Southern CaliforniaLos Angeles, CA, USA; ^11^Department of Veterans Affairs Greater Los Angeles Healthcare SystemLos Angeles, CA, USA

**Keywords:** mouse intra-intestinal model, small bowel, mucus barrier, mucoadhesive particles, mucus penetrating particles

## Abstract

The oral route is the most preferable one when it comes to drug administration. Different animal models have been used to characterize the fate of potential medicines upon oral delivery but fail to clarify specific events occurring at localized sites of the gastrointestinal tract, particularly at the small intestine. We developed a new mouse intra-intestinal infusion model that enabled the direct administration of substances (such as drugs or nanoparticle drug carriers) in the small intestine through an implanted catheter, which can be maintained for prolonged periods of time. The location of catheter insertion can be previously determined as more proximal or distal, allowing to test specific portions of the intestine. Since the model is presumably able to maintain normal physiological characteristics, namely the mucus coating of the intestinal wall, it allowed studying the distribution of different nanoparticles upon localized intra-intestinal administration. The hereby proposed mouse model has the potential to be useful in other types of studies, namely in clarifying localized processes occurring at specific sites of the intestine.

## Introduction

The oral intake of medicines is the main form of drug delivery, making the gastrointestinal tract (GIT) the most important barrier before systemic drug exposure occurs (Mrsny, 2012). Intestinal absorption is one of the main determinants of drug bioavailability, but this essential step for the overall pharmacokinetics is governed by the effective amount of drugs that actually reach the absorptive interface. Indeed, different events occurring between oral intake and intestinal absorption may be determinant in defining the fate of drugs, namely those involved in the release and local distribution of a pharmaceutical dosage form or delivery system (Koziolek et al., 2016). Normal physiological mechanisms (e.g., gastric digestion and emptying, intestinal peristalsis) and barriers (e.g., the mucus covering the mucosal wall, intestinal villi) play key roles in defining localized events at different points of the GIT (McConnell et al., 2008; Mudie et al., 2010). Understanding how these can affect the behavior of drug and/or dosage forms or delivery systems is essential in identifying critical steps that may preclude drug absorption at the intestinal epithelium.

Many animal species and models are currently used for characterizing the overall performance of orally delivered drugs, namely bioavailability, but most fail to characterize focal events at the intestinal mucosa (Musther et al., 2014). The lack of appropriate mechanistic understanding of fundamental steps occurring at pre-absorption stages may limit the ability to effectively modulate the amount of drugs reaching the epithelial surface. This introduces significant bias as to the appropriate measures required for enhancing the bioavailability of molecules of interest and may impair fast and reliable product development, with financial losses thereof.

The mucus layer, in particular, must be taken into account when studying drug bioavailability, since electrostatic interactions, as well as those of hydrophobic nature, between mucins and chemical compounds or dosage forms/delivery systems are of critical importance at the pre-absorption stage (Boegh and Nielsen, 2015).

One of the classical strategies to improve the bioavailability of poorly absorbed drugs or nucleic acids comprises the use of polymer micro- or nano-particles (NPs) as carriers (Gomez-Orellana, 2005). Mucoadhesion due to electrostatic, hydrophobic or van der Walls interactions or inter-polymer chain interpenetration is believed to increase bioavailability in the GIT due to the improvement of the residence time of the particles (Sosnik et al., 2014). Nevertheless, current trend suggests that the effectiveness of these mucoadhesive particles may be limited by the impairment of diffusion across mucus toward the epithelium, followed by the relatively rapid clearance of the superficial luminal mucus (Ensign et al., 2012). Indeed, mucus is considered as a highly effective barrier for the diffusion of large molecules and nanocarriers (Cone, 2009) and may, ultimately, limit absorption. Various *in vitro* and *ex vivo* methods have been developed for studying interactions with mucus but abbreviate many of the features governing *in vivo* events and, thus, lack overall physiological relevance (das Neves et al., 2011; Sigurdsson et al., 2013). Moreover, *in vivo* imaging techniques used for characterizing mucoadhesive properties are unable to provide detailed insights as to the interactions with mucus (Weitschies and Wilson, 2011).

Animal models that allow studying the behavior of nanoparticulate systems at specific sites of the GIT would be welcome in order to overcome the above mentioned limitations. In particular, one that enables the direct instillation of delivery systems at precise locations within the intestine. A mouse intragastric infusion model was previously described by the group of one of the authors of the present study (Ueno et al., 2012), in which a gastrostomy catheter was implanted, allowing for the direct delivery of specific diets or various substances into the stomach. However, and to the best of our knowledge, there are no comparable models that allow the delivery of substances directly into the small bowel. In this article we describe a new mouse model for direct intra-intestinal infusion in order to administer, in a precise way, drugs or delivery systems into specific regions. The feasibility of the model for studying the distribution of different drug nanocarrier surrogates, namely polystyrene (PS)-based mucoadhesive particles (MAP) or mucus penetrating particles (MPP), upon direct administration in the intestinal lumen was also assessed.

## Materials and methods

### Materials

Silicone tubing (SILASTIC®, i.d. 0.30 mm × o.d. 0.64 mm or i.d. 0.51 mm × o.d. 0.94 mm) was purchased from Dow Corning (Auburn, MI, USA), BTPE-25 and BTPE-10 polyethylene tubing (i.d. 0.46 mm × o.d. 0.91 mm and i.d. 0.28 mm × o.d. 0.60 mm) and PinPort™ 25 ga with injector from Instech Laboratories (Plymouth Meeting, PA, USA), Dacron® felt (0.635 mm in thickness) from PEI (Munhall, PA, USA), silicone rubber from Axton (Axton, VA, USA), and RTV catalyst no. 4 from Ellsworth (Germantown, WI, USA). Poloxamer 407 (Kolliphor® P 407, MW 14,600 g/mol) was kindly provided by BASF Corporation (Ludwigshafen, Germany). Red fluorescent carboxylate-modified PS particles (FluoSpheres®) with nominal diameter of 0.2 μm were purchased from Molecular Probes (Eugene, OR, USA). All other materials were of analytical grade or equivalent.

### Animals

Male and female C57BL/6 mice with 8–12 weeks of age obtained from our animal facility were used. Experiments were approved by the ethics committee of the Faculty of Medicine of University of Porto and Direção-Geral de Alimentação e Veterinária (DAGV 0421/000/000/2015). All procedures were conducted according to the European Directive 2010/63/EU on the protection of animals used for scientific purposes and FELASA guidelines.

### Establishment of the animal model

#### Catheter preparation (Figure 1)

A polyethylene tubing (i.d. 0.28 × o.d. 0.60 mm) was cut to a length of 10 mm. A silastic tube (i.d. 0.30 mm × o.d 0.64 mm) was cut to a length of 15 mm, immersed in chloroform in a fume hood for some minutes, to expand the silicone, and slid on one end of the polyethylene tubing.

**Figure 1:**
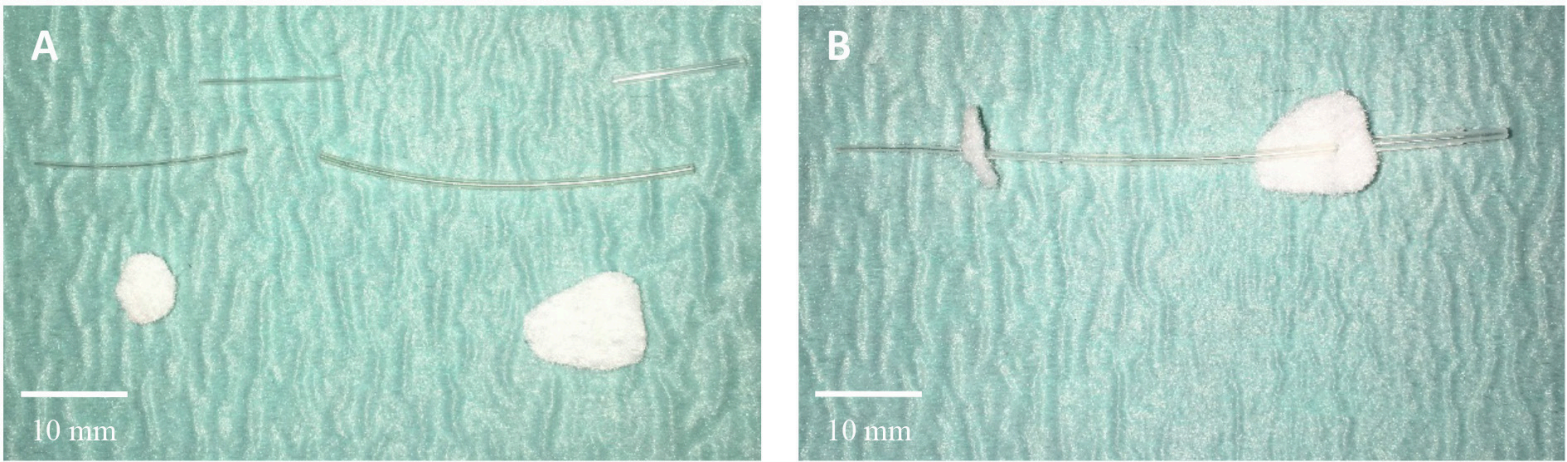
**Preparation of the catheter for intra-intestinal placement. (A)** Catheter components. **(B)** Catheter fully assembled.

A polyethylene tubing (i.d. 0.46 × o.d. 0.91 mm) was cut to a length of 10 mm; a silastic tube (i.d. 0.51 mm × o.d. 0.94 mm) was cut to a length of 40 mm, immersed on chloroform, one of the ends was slid on the remaining end of the thinner polyethylene tube and the other end on the thicker polyethylene tube.

A portion of Dacron felt was cut to a pear shape (8 × 16 mm) and another one to a round shape (6 mm) and a hole was made at the center of each. The catheter was passed through the hole in the pear shaped Dacron and glued with silicone rubber near the connection of the silastic tubes. Then, it passed through the round-shaped Dacron and glued at a distance 10 mm proximal to the tip of the catheter.

#### Surgical technique (Figure 2)

**Figure 2:**
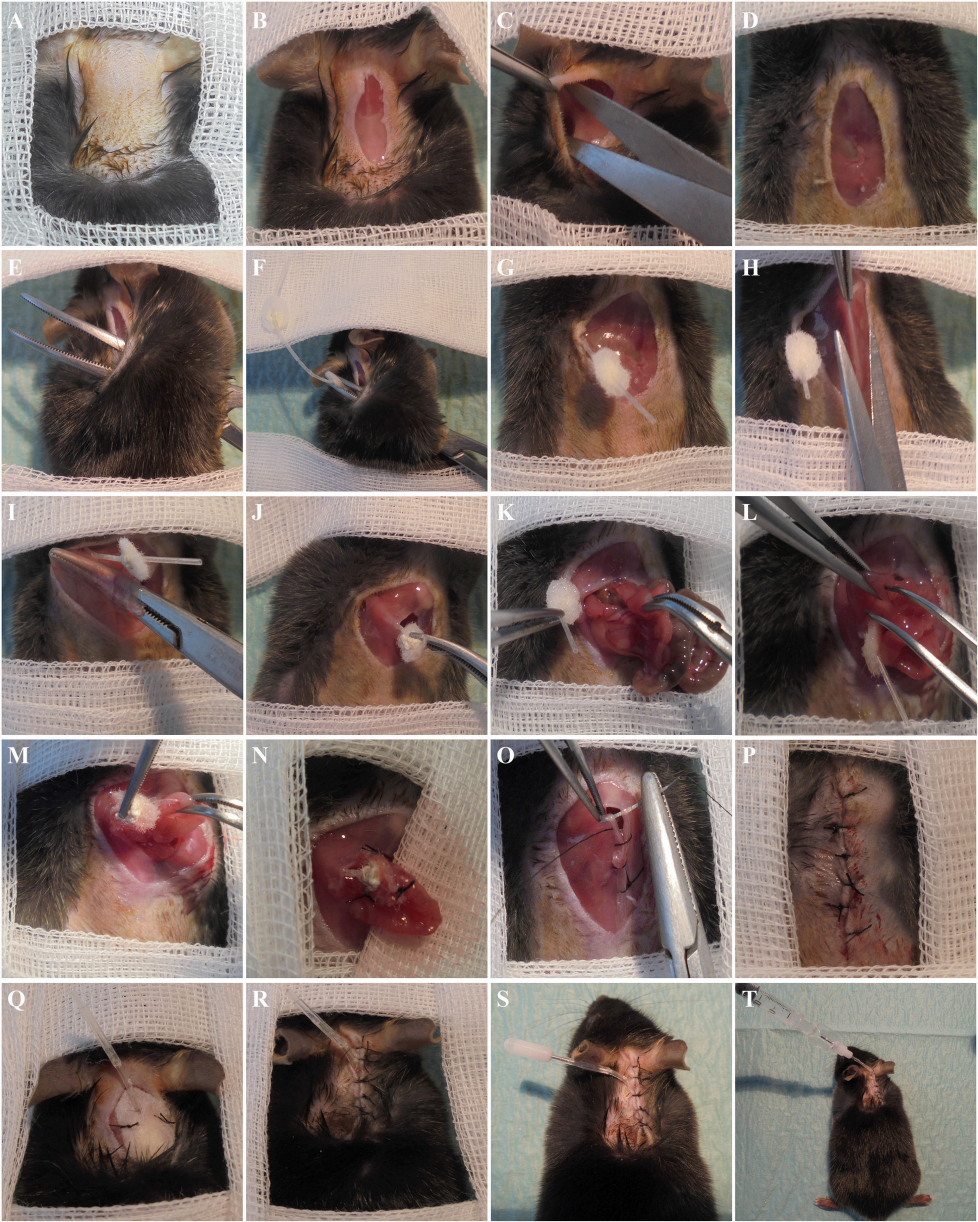
**Surgical procedure used for establishing the proposed mouse model. (A)** Remove hair and swab the dorsum with iodine solution. **(B)** Dorsal midline incision. **(C)** Open/enlarge the lateral plans. **(D)** Ventral incision. **(E)** Insert the curved hemostat through the ventral incision till the dorsal one. **(F)** Hold the catheter in the Dacron disk area. **(G)** Pull out the catheter through the ventral incision. **(H)** Open the visceral peritoneum. **(I)** Make a hole in the peritoneum, pressing a hemostat against the wall in the direction of the right flank. **(J)** Pass the catheter through the hole into the abdominal cavity. **(K)** Expose the small bowel and choose the location to introduce the catheter. **(L)** Puncture the small bowel. **(M)** Insert the catheter tip in the intestine. **(N)** Suture the Dacron disk to the intestine. **(O)** Suture the peritoneum. **(P)** Close the abdominal skin incision. **(Q)** Suture the Dacron to the dorsal muscles. **(R)** Suture the dorsal skin incision. **(S)** Put the PinPort™, 25 ga, at the tip of the catheter. **(T)** Adapt the injector to the PinPort™.

Mice were anesthetized with intraperitoneal ketamine (80–100 mg/kg) and xylazine (10 mg/kg). Hair from the dorsal neck (Figure 2A) and mid-abdomen was shaved and hairless areas were swabbed with povidone-iodine solution. Ointment was applied in mouse's eyes and the tongue was pulled out. The mouse was placed onto a heating pad covered with a sterile surgical towel on ventral decubitus, to avoid hypothermia, and the areas of the body not prepared were covered with sterile gauze.

An incision was made in the skin of the dorsum (around 10 mm) in the interscapular region (Figure 2B) and we used a scissor to separate the skin and the muscle tissue from the incision site to the right flank, in order to create a subcutaneous tunnel (Figure 2C). The mouse was placed in dorsal decubitus and an incision was made in the midline of the abdomen (around 15 mm), beneath the xiphoid cartilage (Figure 2D), and a scissor was used to separate the skin from the muscle, moving it to the right flank, in order to complete the subcutaneous tunnel started in the dorsum.

The mouse was placed on its left side and a curved hemostat was inserted from the abdominal incision through the subcutaneous tunnel till the dorsal incision (Figure 2E), the catheter grasped with the hemostat (Figure 2F) and the tip pulled to the abdominal incision (Figure 2G). The mouse was then put in dorsal decubitus and a longitudinal incision was made in the linea alba in order to open the peritoneal cavity (Figure 2H). A curved hemostat was put into the abdominal cavity, the tip pressed against the abdominal wall approximately 15 mm toward the right flank and an aperture was made (Figure 2I). The catheter tip was grasped and pulled into the abdominal cavity, using the hemostat (Figure 2J).

Afterwards, the small intestine was gently exposed, starting in the distal ileum (by identifying the cecum) through the proximal jejunum (Figure 2K), and the site to make a small opening was chosen, using a jeweler's forceps (Figure 2L). The exposed bowel was hydrated with sterile saline (around 0.5 mL). The tip of the catheter was inserted into the small bowel and the catheter was held with a curved forceps (Figure 2M). A 6/0 silk thread was used to suture the Dacron disk to the intestinal wall (3–4 stitches) (Figure 2N). Twenty-five to fifty micro liter of saline were infused into the small intestine through the catheter to exclude any leakage.

The whole intestine was placed in its original position, and the catheter was gently pulled from the dorsal side. The peritoneal cavity was closed with 6/0 silk thread in a continuous suture (Figure 2O) and the abdominal skin with silk thread in an interrupted suture (Figure 2P).

The mouse was placed in ventral decubitus and the pear-shape Dacron positioned along the muscle, sutured to it with silk thread (Figure 2Q) and, finally, the dorsal skin was closed with silk thread in an interrupted suture (Figure 2R).

The mouse was placed in a cage warmed by an infrared light lamp and an analgesic (tramadol 20 mg/Kg, IP) was administered as the animal began to recover from anesthesia. Analgesia continued during the following 2 days if there was any sign of pain. A 3-day antibiotic course (enrofloxacin 5 mg/Kg/day, IP) was also performed starting in the day of surgery.

Finally, the PinPort™25 ga was adapted to the end of the catheter (Figure 2S) and the injector was employed to adapt a syringe to the PinPort™ (Figure 2T).

### Preparation and characterization of nanoparticles

Unmodified FluoSpheres® were used as mucoadhesive particles (MAP). Mucus penetrating particles (MPP) were obtained by surface modification (adsorption) of the previous with ~5.7 kDa poly(ethylene oxide) (PEO). Briefly, FluoSpheres® were incubated overnight at a concentration of 0.2% (*w/v*) in 1% (*w/v*) poloxamer 407 aqueous. Obtained PEO-modified NPs were then filtered by centrifugation at 167 × *g* using an Amicon® filter tube (MWCO = 100 kDa, Millipore Corporation, Bedford, MA, USA) in order to wash excess poloxamer and concentrate the particles. The same procedure was used for unmodified NPs (i.e., FluoSpheres® without PEO coating) but ultra-pure water was used instead of poloxamer 407 solution during incubation.

NPs were characterized regarding hydrodynamic diameter and size distribution by dynamic light scattering, and zeta potential by laser Doppler anemometry using a Zetasizer Nano ZS (Malvern Instruments, Worcestershire, UK). NPs were diluted at approximately 0.02% (w/v) in 10 mM NaCl solution and all measurements were performed in triplicate at 25°C.

### Nanoparticle administration, intestinal tissue collection and processing, and confocal microscopy assessment

Mice were used 1 week after the surgical procedure to allow reestablishment of the normal physiologic status. Mice were kept on a liquid diet for 24 h followed by 24 h without any food access in order to reduce the consistency of stool. Then, the intestinal lumen was flushed with 50 μL normal saline via the catheter and mice were allowed to rest for 1 h to reconstitute the mucus layer.

One hundred micrograms of NPs were diluted in 50 μL of deionized water and administered through the intestinal catheter. Mice were sacrificed after 1 h and sections of the jejunum were collected and frozen in O.C.T. Compound (Shandon Cryomatrix™, Thermo Scientific, Runcorn, Cheshire, UK). Cross-sectional cuts of 10 μm thickness were performed using a Leica CM3050 S cryostat (Leica Biosystems, Germany), mounted in microscope slides, fixed briefly in 10% formalin, air dried, and mounted using Vectashield® with 4′, 6-diamidino-2-phenylindole (DAPI) (Vector Laboratories, Inc, USA). Images were obtained using a Leica TCS SP5II confocal microscope (Leica Microsystems, Germany).

## Results and discussion

Although several *in vitro, ex vivo* or *in situ* methods for studying intestinal absorption and other associated phenomena (e.g. drug carrier transport) are widely used (Sarmento, 2016), data obtained from these last must be interpreted carefully, since some of the physiologic conditions of the normal intestine are altered. Thus, the development of models that more closely mimic the *in vivo* situation is desirable. In particular, the mucus layer differs along the GIT (Atuma et al., 2001; Sadio et al., 2014) and must be taken into account while studying transport and absorption processes (Boegh and Nielsen, 2015). However, most non-*in vivo* models fail to maintain the integrity or even consider the presence of mucus. Also, current *in vivo* animal models can only measure the outcome of drug absorption, typically translated as systemic pharmacokinetics monitoring, thus limiting the ability to fully understand pre-absorption events that are known to play key roles in the fate of drugs and dosage forms/delivery systems in the gut (Musther et al., 2014).

In order to address these issues, we modified a previously reported mouse intragastric infusion model (Ueno et al., 2012) and developed a new one in which test substances/materials can be directly delivered to the small bowel, through a permanently implanted catheter.

A previously prepared catheter was introduced directly in the jejunum. Due to the small size of the lumen, the amount of time required for the procedure was longer than that required for insertion of the catheter in the stomach (~ 45–50 min). Special attention was paid to the direction of the catheter during insertion, which must be pointing toward the cecum (Figure 2K). So, in order to avoid wrong positioning and consequent intestinal obstruction, the whole small bowel should be exposed, thus allowing to identify the cecum and assuring that the catheter is correctly implanted. Antibiotics were administered for 3 days, starting at the day of the surgery in order to prevent abdominal infections. It is required that the animals are isolated in individual cages to preclude chewing of the catheter by their mates. Mice resumed normal diet on the day of the surgery and were allowed to recover for 1 week before any further experiments being conducted. Flushing with 50 μL of saline was performed every day to prevent clogging of the catheter lumen. Mice presented normal behavior upon surgery and the catheter could be kept for at least 4–6 weeks. Animal death rate was around 10% after the surgical procedure was fully established. Although all the dead mice were subjected to necropsy, no apparent anomalies were macroscopically detected, namely signs of infection.

The proposed animal model may potentially hold several advantages over the most commonly established *in vitro* and *ex vivo* models, mainly because the intestine is maintained in normal physiological conditions and tissue integrity is preserved. Hence, a fully functional intestinal barrier and an intact blood supply and nervous systems allow complete mimicking of the normal transit, digestion and absorption dynamics of the gut. The catheter can be maintained for prolonged periods of time and allow multiple administrations without the need to sacrifice the animals. Besides, the rate and mode of administration of food or drugs/delivery systems can be precisely controlled, both in terms of timing and amount. Another advantage of this model is the possibility to choose the place where the catheter will be inserted, which can be an added value to evaluate intestinal permeability to drugs or performance of delivery systems at particular foci. One example of studies where this model could be employed is in the development of controlled-release systems to target proximal (jejunum) or distal (ileum) parts of the small bowel. It can also be used in cases where avoidance of the gastric emptying is required.

Besides, the possibility to administer low volumes of fluids is an advantage over the close loop models, whose results can be inflated due to the high pressure and the disruption of the mucus barrier (Reineke et al., 2013). The results can also be confusing depending on the volumes administered by oral gavage (Eyles et al., 1995).

In the present work, we wanted to show the value of the intra-intestinal model in studying the behavior of NPs in the intestinal lumen and provide insights as to the distribution of MAP and MPP. Previous studies have shown that MAP administered by oral gavage in high volumes or administered in a close intestinal loop model have a similar distribution, in small intestine, as MPP. In opposition, when administered in low volumes by oral gavage, MPP distributed evenly in the tissue, while MAP were clumped in the lumen (Maisel et al., 2015).

We used unmodified carboxylate FluoSpheres® and PEO-modified counterparts as MAP and MPP, respectively. PEO-modified fluorescent NPs were obtained by surface adsorption of the tri-block PEO-PPO-PEO poloxamer 407 as previously described (Tang et al., 2009). The PPO middle section of the copolymer is hydrophobic and can adsorb to the PS surface of NPs, while exposing both PEO hydrophilic arms outwards to the aqueous medium (Shenoy and Amiji, 2005). This reversible modification of the surface confers a highly hydrophilic, non-charged corona to the particles, which prevents interactions with mucin fibers and renders high mobility in mucus as demonstrated *in vitro* (Wang et al., 2008; Tang et al., 2009). This behavior has been shown particularly relevant in providing enhanced distribution and uniform coating of mucosal surfaces when mucus-inert NPs were administered to mice by the oral, rectal or vaginal routes (Cu et al., 2011; Maisel et al., 2015; Xu et al., 2015).

Main size and surface charge features for both plain and PEO-modified FluoSpheres® are presented in Table 1. Values of hydrodynamic diameter were in range with those reported by the manufacturer (0.2 μm). A slight increase in size was observed after PEO-modification, which can be attributed to the poloxamer coating at the surface of NPs. Both type of NPs were monodisperse, as assessed by the results for the polydispersity index (PdI). As anticipated, major differences were observed for zeta potential. Contrasting with unmodified particles, which presented markedly negative values for zeta potential due to the surface presence of carboxyl groups, near neutral values were obtained for PEO-modified NPs. This indicates that dense coating with poloxamer was achieved, thus shielding the surface charge of FluoSpheres® (Yang et al., 2011).

**Table 1:** **Hydrodynamic diameter, polydispersity index (PdI) and zeta potential of MAP and MPP**.

**NP type**	**Hydrodynamic diameter (nm)**	**PdI**	**Zeta potential (mV)**
MAP	182 ± 1	0.028 ± 0.013	−45.0 ± 1.9
MPP	196 ± 1	0.027 ± 0.005	−4.6 ± 0.2

*Results are expressed as mean± standard deviation (n = 3)*.

**Figure 3:**
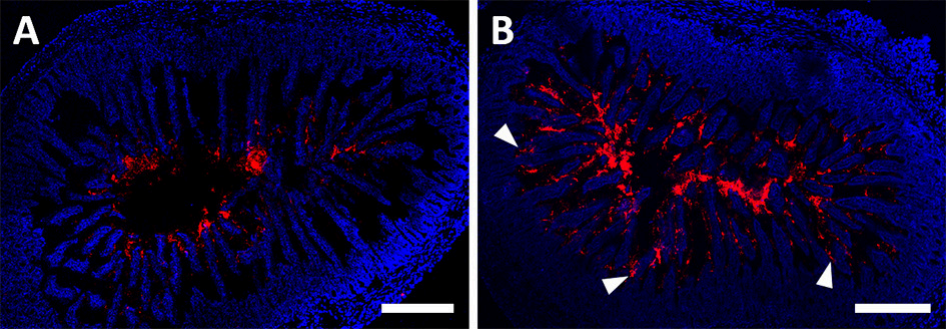
**Distribution of NPs in the jejunum at 1 h after administration**. Distribution of MAP **(A)** and MPP **(B)** in the jejunum after administration through the intra-intestinal catheter. Red and blue signals are from particles and cell nucleus (DAPI staining of chromosomic DNA), respectively. Arrow heads highlight some examples of nanoparticle clusters deeply embedded in between villi. Scale bar 300 μm. Images are representative of three mice.

In the present study MAP or MPP were administered to the developed animal model and confocal fluorescence microscopy of the excised jejunum was performed. Imaging was able to successfully track the position of NPs within the gut (Figure 3). In particular, microscopy studies evidenced that MPP were able to more evenly distribute throughout the jejunum as compared with MAP. PEO-modified NPs migrated deeply into intestinal villi thus evidencing their muco-inert nature, while plain FluoSpheres® were mainly retained within the central lumen, presumably due to the strong interaction with mucus. Although the intention of this work was not to compare different possibilities for delivery routes of NPs, our results are parallel to those reported in a previous study where NPs were administered by oral gavage in low volumes to mice (Maisel et al., 2015). In particular, data from our model in tandem with this last study seems to reinforce that the behavior of PEO-modified NPs is not affected by gastric residence (as would occur upon gavage), assuming that poloxamer adsorption is stable enough to withstand the harsh environment of the stomach. Since the amount of NPs reaching the region of interest was complete in the case of our model, it seems plausible to assure that the results previously reported for PEO-modified particles were not biased by the possibility of pre-jejunal sorting and retention of a population of adhesive particles. This information from focal analysis of NP transport at the jejunum may also be important in cases when poor performance of delivery systems is indeed affected by gastric residence.

## Conclusion

In this work we developed a new mouse intra-intestinal infusion model and used it successfully for studying the distribution of different type of NPs upon direct administration into the jejunum. Results confirmed previous data that MPP are able to better penetrate intestinal folding in between villi as compared to MAP. We believe that the proposed model may further be useful for several other applications, namely in identifying specific sites for drug absorption, characterizing the intra-intestinal metabolism of drugs and assess focal toxicity of drugs and delivery systems, to name a few.

## Author contributions

Conceived and designed the experiments: AS, RN, BS, RA, HT, JdN. Performed the experiments: AS, AA, RN, SR. Analyzed the data: AS, AA, RN, JdN. Wrote the manuscript: AS, RA, JdN. Revised the manuscript: BS, RA, HT.

## Funding

This work was co-financed by Sociedade Portuguesa de Gastrenterologia, Fundação para a Ciência e a Tecnologia (FCT), Portugal (VIH/SAU/0021/2011), by FEDER—Fundo Europeu de Desenvolvimento Regional funds through the COMPETE 2020—Operacional Programme for Competitiveness and Internationalisation (POCI), Portugal 2020, and by Portuguese funds through FCT—Fundação para a Ciência e a Tecnologia/Ministério da Ciência, Tecnologia e Inovação in the framework of the project “Institute for Research and Innovation in Health Sciences” (POCI-01-0145-FEDER-007274). This work was also financed by the project DOCnet (NORTE-01-0145-FEDER-000003) supported by Norte Portugal Regional Programme (NORTE 2020), under the PORTUGAL 2020 Partnership Agreement, through the European Regional Development Fund (ERDF). The Gulbenkian Programme for Advanced Medical Education was sponsored by Fundação Calouste Gulbenkian, Fundação Champalimaud, Ministério da Saúde and FCT. Rute Nunes and José das Neves gratefully acknowledge FCT for financial support (grants SFRH/BD/96519/2013 and SFRH/BPD/92934/2013, respectively). Training in catheter construction and implantation was provided by the Animal Core of the Southern California Research Center for ALPD and Cirrhosis funded by a NIAAA/NIH center grant P50AA011999.

### Conflict of interest statement

The authors declare that the research was conducted in the absence of any commercial or financial relationships that could be construed as a potential conflict of interest.
